# Efficacy Analysis of Arthroscopic Treatment of Synovial Chondromatosis of the Knee: A Retrospective Study of More Than Five Years

**DOI:** 10.1111/os.70132

**Published:** 2025-07-21

**Authors:** Yang Xu, Tao Li, Wenjing Ma, Lei Yao, Jian Li

**Affiliations:** ^1^ Sports Medicine Center, West China Hospital Sichuan University Chengdu China; ^2^ Department of Orthopedics, Orthopedic Research Institute, West China Hospital Sichuan University; Chengdu China

**Keywords:** arthroscopy, clinical efficacy outcomes, long‐term outcome, osteoarthritis, synovial chondromatosis

## Abstract

**Objective:**

The synovial chondromatosis is an exceptionally rare benign condition, predominantly found in the knee joint, and can result in pain, restricted mobility, and potential irreversible damage to the joint and cartilage. Despite the utilization of arthroscopic techniques in the surgical management of synovial chondromatosis, there remains a paucity of long‐term assessment regarding its efficacy. The main objectives of this study include: (i) investigating the long‐term efficacy of arthroscopic surgery in patients with knee synovial chondromatosis;(ii) identifying factors influencing functional improvement in patients post‐surgery function.

**Methods:**

We conducted a retrospective analysis of all patients with synovial chondromatosis of the knee who underwent arthroscopic synovectomy and loose body removal at our institution between June 2009 and January 2020. The follow‐up period for all cases exceeded 5 years. Data collection included demographic details, clinical efficacy indicators(VAS, KOOS, WOMAC, etc.), imaging findings, and subjective satisfaction of patients with surgical outcomes. Data analysis selected *t*‐tests, ANOVA, non‐parametric tests, and correlation methods based on normality test results.

**Results:**

We enrolled a total of 13 patients, including 4 men and 9 women, with a mean follow‐up of 113.15 ± 30.45 months (range 61–145). There were no postoperative complications, recurrence, or malignant transformation in all patients, and the VAS scores, KOOS scores, WOMAC scores, and Lysholm scores of all patients were significantly improved at 3 months, 6 months, 1 year, 5 years, and the last follow‐up (*p* < 0.05). However, one patient experienced osteoarthritis progression, necessitating arthroplasty.

**Conclusion:**

This retrospective study demonstrated that arthroscopic treatment for knee synovial chondromatosis is effective and safe. It leads to immediate post‐intervention improvement in symptoms and function, with sustained long‐term benefits.

## Graphical abstract

With more than 5 years of follow‐up, we found arthroscopic‐free body resection and synovectomy to be a safe and effective method of treating patients with synovial chondromatosis of the knee. All patients experienced significant postoperative pain relief and functional improvement, which were maintained during long‐term follow‐up; patient age, time of onset, gender, side of onset, and imaging grading had no significant effect on outcome.

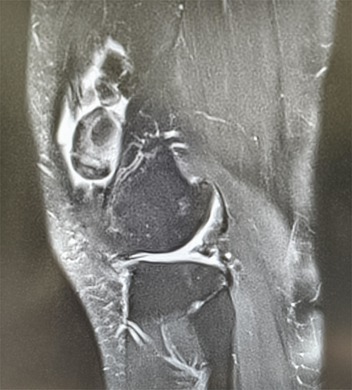

## 1 Introduction

Synovial chondromatosis (SC) is a rare, benign disease that usually occurs in people aged 30–60 and affects more men than women [1, 2]. The exact incidence of synovial chondromatosis is unknown, but a previous study reported it affects 1.8 million people yearly [3]. SC often occurs in a single joint, with the knee having the highest prevalence of 60%–70%, but some patients develop it bilaterally, with a prevalence of around 10% [4, 5, 6].

The pathogenesis of SC is thought to be probably related to chemotaxis, which is most clearly characterized by the development of focal cartilage within the synovium and the formation of cartilaginous nodules, which can detach from the synovial breaks and form loose bodies within or around the joint [7, 8]. These loose bodies can then receive nutrients from synovial fluid and continue to grow, reattach to the synovium, or be resorbed. If the escape of loose bodies occurs through the joint capsule, extra‐articular involvement may result [5, 9]. Primary SC is rarer and occurs earlier, with no clear association with trauma or infection [8, 10]. Secondary SC, conversely, is more common and may result from a single trauma or repeated mechanical irritation, and is usually accompanied by osteoarthritis, exfoliative osteochondritis, synovitis, and other diseases [11, 12, 13]. At the same time, the presence of loose bodies may lead to increased progressive wear of the articular surface and increase the degree of osteoarthritis in patients [14]. Some other scholars believe that SC belongs to a tumor, but no conclusive evidence has been obtained yet [1].

Although SC is a benign disease, the production of these loose bodies can lead to clinical symptoms such as pain, swelling, hinging, and limited joint mobility, which can seriously affect the patient's quality of life and, when the disease progresses to an advanced stage, may lead to joint replacement [15, 16, 17]. Malignant transformation also occurs in some SC patients, but the incidence is low, approximately 5%, and may be associated with recurrence [9, 18, 19, 20].

Although conservative treatments (e.g., taking nonsteroidal anti‐inflammatory drugs) can control symptoms such as pain and swelling, surgical treatment becomes an integral part of the intervention program when the disease progresses [2, 21]. Traditional open surgery exposes more of the surgical field and thus may have a better chance of eradicating the disease [10, 20]. In previous studies, open surgery has demonstrated comparatively favorable therapeutic outcomes; however, it is not without its shortcomings, including significant trauma and an extended recovery period [22, 23]. In recent years, arthroscopic techniques have also been increasingly utilized in the surgical treatment of SC. The advantages of arthroscopic surgery in treating SC are mainly due to smaller surgical incisions, less postoperative pain, and early functional recovery [4, 19, 24]. Although the knee joint is the joint with the highest incidence of SC, there are few clinical studies on the use of arthroscopic technology to treat SC, and there is a lack of long‐term evaluation of the effectiveness of arthroscopic technology.

In this study, we retrospectively analyzed patients with synovial chondromatosis of the knee diagnosed at our tertiary referral center in China between 2009 and 2020, and comprehensively evaluated the efficacy of arthroscopic surgery for the treatment of synovial chondromatosis of the knee in long‐term follow‐up by various aspects, such as knee function scores, pathological findings, and imaging indexes. The main objectives of this study include: (i) investigating the long‐term efficacy of arthroscopic surgery in patients with knee synovial chondromatosis; (ii) identifying factors influencing functional improvement in patients post‐surgery function.

## 2 Patients and Methods

### 2.1 Study Design

We retrospectively studied patients with synovial chondromatosis of the knee who underwent arthroscopic‐free body resection and synovectomy at the Sports Medicine Center of a tertiary referral hospital in western China from June 2009 to January 2020. This study has been approved by the Biomedical Ethics Review Committee of West China Hospital of Sichuan University with approval number 2023 (2398). Informed consent for publication of individual clinical details and images was obtained from all study participants. After undergoing arthroscopic surgery, all patients receive routine nursing care and engage in rehabilitation training. They are also administered postoperative preventive anti‐inflammatory treatment. The rehabilitation program involves progressively increasing the range of motion and strengthening the muscles surrounding the joints.

### 2.2 Inclusion and Exclusion Criteria

The inclusion criteria for the studies were as follows: (1) 18–85 years of age; preoperative diagnosis of synovial chondromatosis of the knee after physical examination and imaging and intraoperative confirmation of the diagnosis; (2) poor efficacy of non‐surgical treatments (including non‐steroidal anti‐inflammatory drugs, rest, etc.); (3) arthroscopic resection of synovial chondromatosis and synovectomy of the knee joint were performed; postoperative rehabilitation was completed; (4) patients with a follow‐up period of more than 5 years; (5) patient's agreement to regular follow‐up.

**FIGURE 1:**
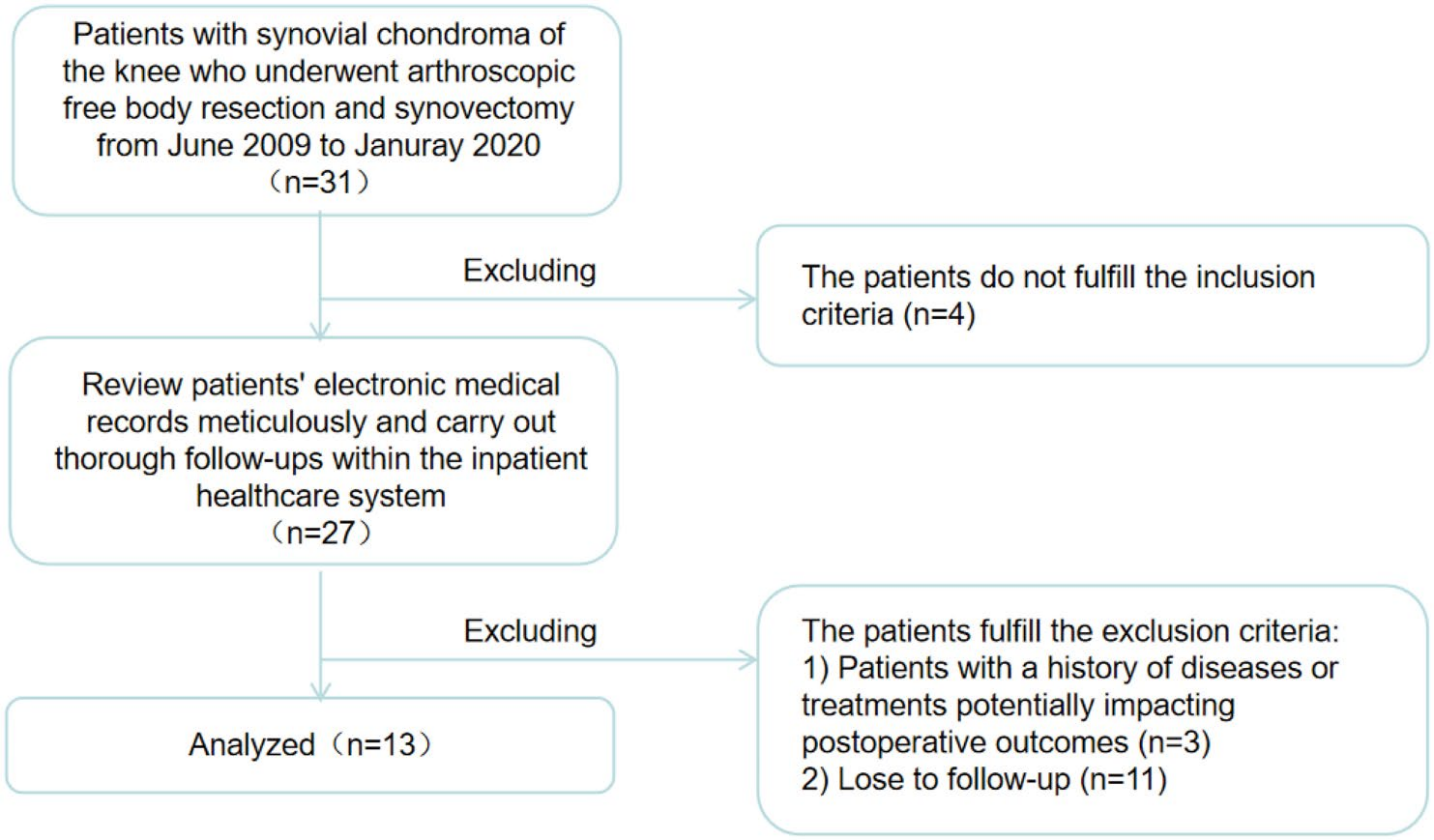
The inclusion and exclusion process for patients.

The exclusion criteria for studies were as follows: (1) Patients with a history of diseases or treatments potentially impacting postoperative outcomes include those with a history of active knee infections, chronic osteomyelitis, or septic arthritis; autoimmune diseases (e.g., rheumatoid arthritis), metabolic bone diseases (e.g., hyperparathyroidism), severe osteoporosis, or gouty arthritis; a combined history of ipsilateral knee joint fracture, malignant bone tumor, or local radiotherapy; and long‐term use of glucocorticoids, immunosuppressants, or anticoagulant therapy. (2) Patients who have undergone knee joint interventions and may present with confounding factors are those whose affected knee joint has previously undergone open surgery, arthroscopy, synovectomy, or intra‐articular injection therapy (such as steroids). (3) Patients who are unable to cooperate with postoperative rehabilitation plans or are lost to follow‐up will be excluded from the study. The inclusion and exclusion process for patients is shown in Figure 1.

### 2.3 Data Collection

The follow‐up period was preoperative, 3 months, 6 months, 1 year, and 5 years postoperatively, and the final follow‐up was conducted in February 2025. The data collected included demographic characteristics, clinical efficacy indicators, imaging data, and histopathology. The extracted and summarized data included the (1) *demographic characteristics*: gender, age, duration of follow‐up, and side of injury; (2) *clinical efficacy indicators*: recurrence, postoperative complications, malignant transformation, knee joint range of motion, visual analog scale (VAS), Knee Injury and Osteoarthritis Outcome Score (KOOS), Western Ontario and McMaster Universities Osteoarthritis Index Scale (WOMAC), Lysholm Knee Score; (3) subjective satisfaction of patients with surgical outcomes; (4) *imaging indicators*: patients routinely undergo preoperative knee X‐rays and knee MRI to facilitate patient assessment. In this study, the Kellgren and Lawrence criteria were used to grade the degree of osteoarthritis of the knee in patients; the Recht criteria were used to grade the degree of damage to the articular cartilage.

### 2.4 Statistical Analysis

SPSS 22.0 statistical software was used to analyze the data, and descriptive epidemiological methods were used for analysis. The measurements were first subjected to the Shapiro–Wilk test for normal distribution. The *t*‐test was used to compare the measurement data. The χ^2^ test or Fisher's exact test was used to compare the count data; data that do not follow a normal distribution are tested using nonparametric tests. The difference was considered statistically significant when *p* < 0.05 was considered the same as *p* < 0.05 between groups. When *p* < 0.05, the difference between groups was supposed to be statistically significant. We will conduct a correlation analysis to compare the relationships between various factors, including age, onset time, and changes in pain and functional scores (Δ). To quantify the linear relationship between two continuous variables, we will utilize the Pearson correlation coefficient. For data that does not adhere to a normal distribution, we will opt for the Spearman correlation coefficient to capture the association. Additionally, when analyzing the relationship between ordered categorical variables and continuous variables, we will employ the Spearman correlation coefficient. Furthermore, to assess the relationship between ordered categorical variables and continuous variables, we will use either the analysis of variance or the Kruskal‐Wallis test, depending on the data characteristics. Multiple comparisons use Tukey's method to control the Type I error rate.

## 3 Result

### 3.1 Patient Baseline Characteristics and Clinical Outcomes

**FIGURE 2:**
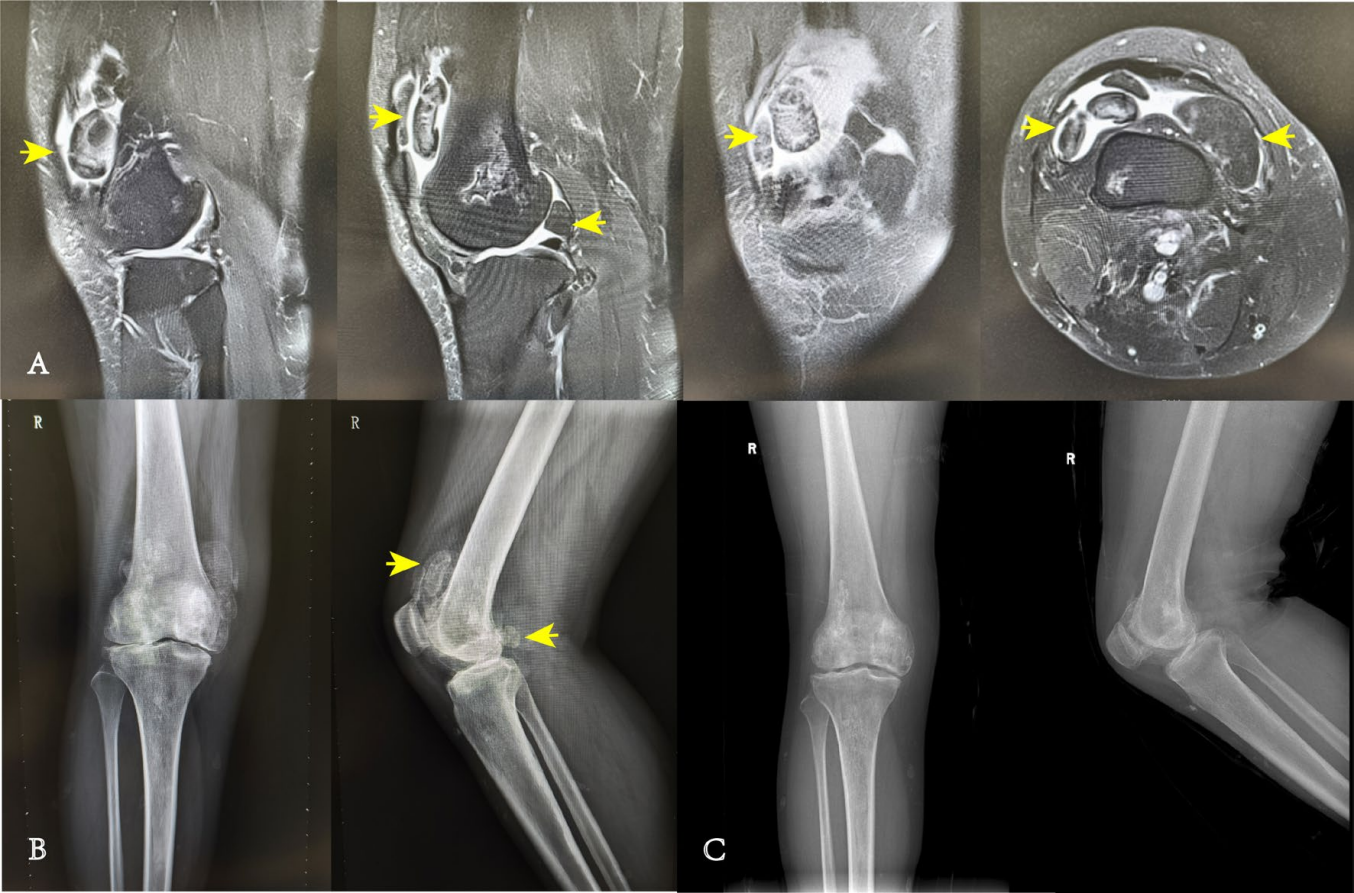
The patient is a 36‐year‐old female who presented with pain and discomfort in her right knee for over 8 years, which had worsened in the past 2 months. Physical examination revealed significant swelling of the right knee, mild tenderness, notable patellar crepitus, and a palpable mass in the suprapatellar pouch. During surgery, extensive hyperemia and thickening of the synovium were observed. Multiple large, smooth synovial chondromatosis nodules of varying sizes were removed from the suprapatellar pouch. Additionally, several broad bean‐sized loose bodies were found in the upper part of the suprapatellar pouch, the posterolateral compartment, and the intercondylar fossa. Pathological examination showed synovial tissue hyperplasia with calcification. Postoperatively, the patient's symptoms improved significantly, and her function recovered well. (A) The patient's MRI shows multiple loose bodies within the knee joint; (B) the patient's X‐ray reveals knee synovial chondromatosis and osteoarthritis; (C) the patient's X‐ray at the last follow‐up. The yellow arrows indicate the loose bodies in the suprapatellar pouch of the knee.

**FIGURE 3:**
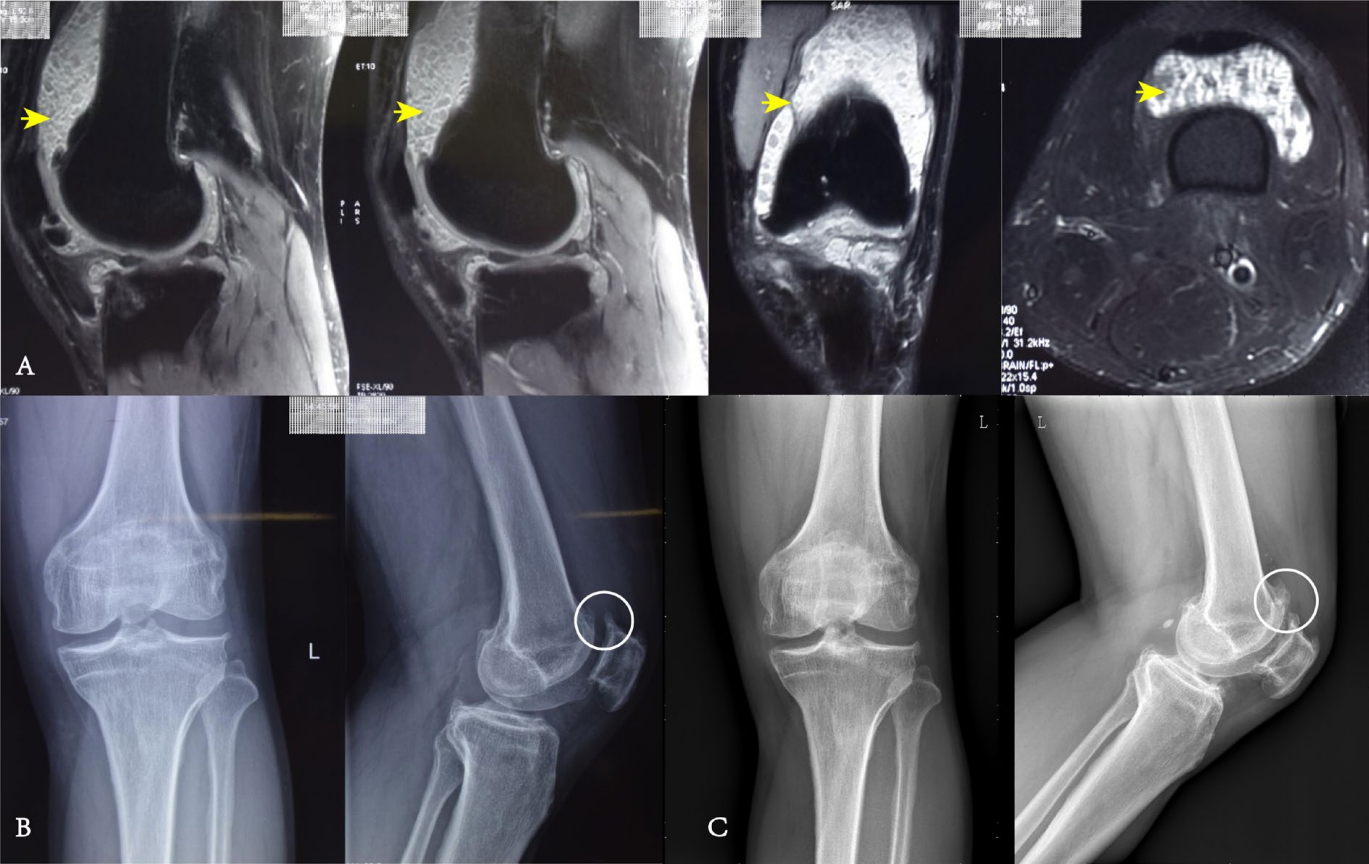
The patient is a 53‐year‐old male who presented with left knee pain accompanied by locking for 6 years. Physical examination revealed tenderness in the left knee, significant patellar crepitus, and marked limitation of movement. During surgery, mild synovial hyperplasia was observed, along with obvious degeneration of the articular cartilage on the femoral condyles and patella. Several hard, variably sized loose bodies were found within the joint space. Pathological examination showed bone/cartilage components consistent with synovial chondromatosis. Postoperatively, the patient's symptoms improved significantly, and his function recovered well. (A) The patient's MRI shows multiple loose bodies within the knee joint; (B) the patient's X‐ray reveals secondary osteoarthritis in the knee joint, with significant osteophyte formation in the patellofemoral joint; (C) the patient's X‐ray at the last follow‐up. The yellow arrows indicate the loose bodies in the suprapatellar pouch of the knee, and the white circles mark the osteophytes.

A total of 13 patients (4 males; 9 females) who met the inclusion–exclusion criteria were enrolled in our study during the period from June 2009 to January 2020, with a mean follow‐up time of 113.15 ± 30.45 months (range 61–145), a mean time to onset of illness of 47.00 ± 34.10 months (range 2–96), a mean age of 44.32 ± 16.10 (range 18.2–63.8) years, left‐sided onset in eight patients, right‐sided onset in five patients, and bilateral onset in one patient. None of the patients had postoperative infections or neurologic or vascular complications, and none had malignant transformation and no recurrence of clinical symptoms. The Kellgren and Lawrence criteria graded the patients. A total of seven patients were grade 1, two patients were grade 2, four patients were grade 3, and there were no grade 4 patients. Using the Recht criteria for grading cartilage injuries, there were three patients with grade 1, four patients with grade 2, three patients with grade 3, three patients with grade 4, and no patients with grade 5 (Table S1). Typical cases are shown in Figures 2 and 3.

### 3.2 VAS

**FIGURE 4:**
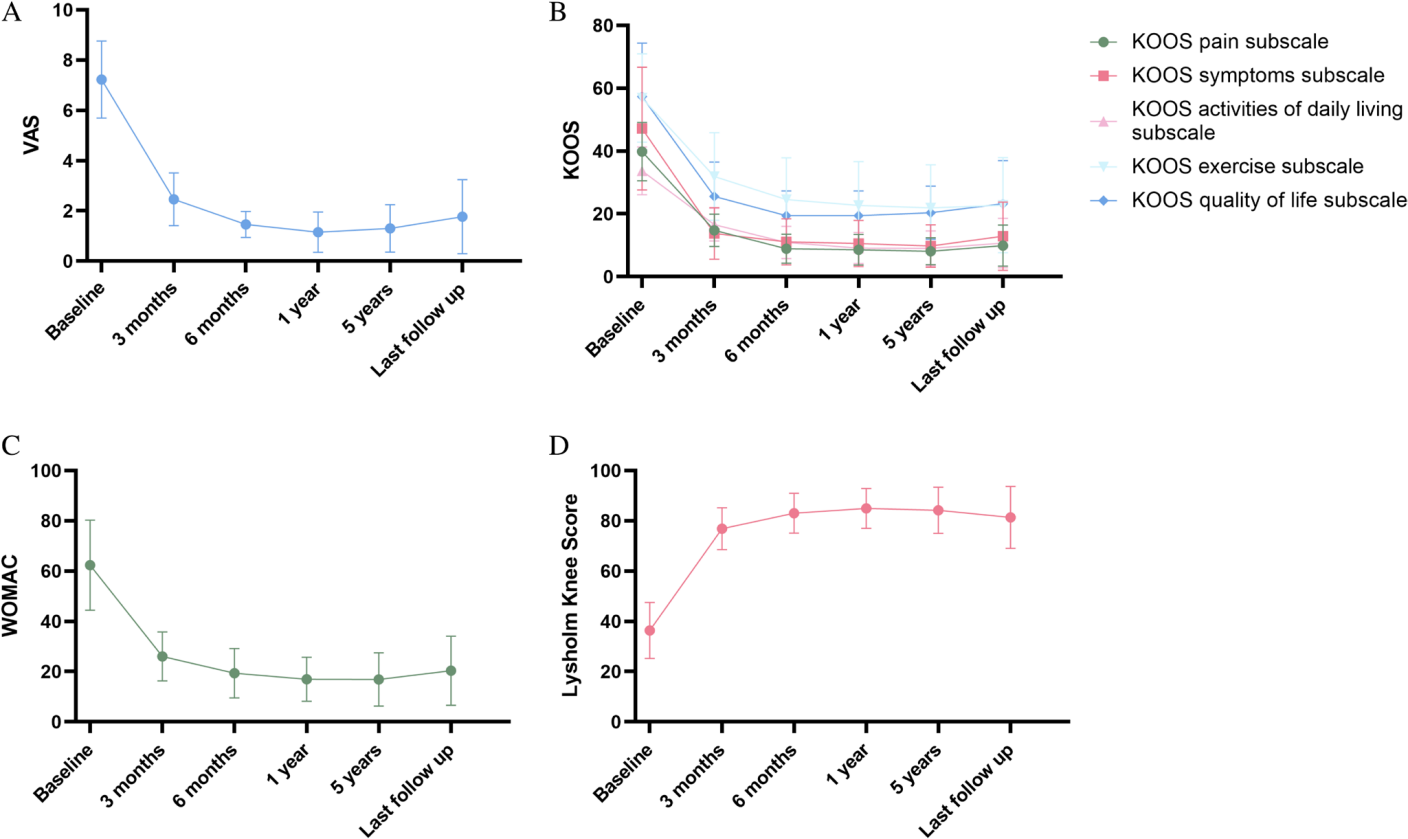
(A) Changes in VAS scores of patients; (B) changes in KOOS score of patients; (C) changes in WOMAC score of patients; (D) changes in Lysholm Knee Score of patients. KOOS: Knee Injury and Osteoarthritis Outcome Score; VAS: visual analog scale; WOMAC: Western Ontario and McMaster Universities Osteoarthritis Index Scale.

Postoperatively, VAS decreased over time until the last follow‐up, when the mean pain score decreased significantly from 7.23 ± 1.54 preoperatively to 1.77 ± 1.48, with statistically significant improvements in pain at all follow‐up time points compared with preoperatively (Table S2 and Figure 4A). Four patients with elevated VAS scores were seen at the final follow‐up and three were over 50. Correlation analysis showed no significant correlation between ΔVAS and age, gender, follow‐up time, side of onset, or imaging grading. The ΔVAS was positively correlated with the onset time (*p* < 0.05).

### 3.3 KOOS

Compared with the preoperative period, all five subscales of KOOS showed significant improvement, with the KOOS pain subscale decreasing from 39.85 ± 9.31 to 9.52 ± 6.59, the KOOS symptom subscale decreasing from 47.15 ± 19.55 to 12.92 ± 7.87, the KOOS activities of daily living subscale decreasing from 33.69 ± 7.58 to 10.69 ± 7.89, KOOS exercise subscale decreased from 56.92 ± 14.07 to 22.78 ± 15.13. KOOS quality of life subscale decreased from 57.38 ± 17.02 to 23.23 ± 13.71 (Table S3 and Figure 4B). At the final follow‐up, the pain, symptoms, ability to perform activities of daily living, sports and recreation, and quality of life subscales of KOOS showed elevated scores in six, four, five, three, and four patients, respectively, who were older than 50 years. Correlation analysis reveals that there is a statistically significant positive correlation between the onset time and the scores of both the ΔKOOS exercise subscale and the ΔKOOS quality of life subscale (*p* < 0.05). Additionally, the scores of the ΔKOOS exercise subscale and the ΔKOOS symptom subscale exhibit a relationship with gender (*p* < 0.05). However, no correlated factors were identified in the other sections of the ΔKOOS.

### 3.4 WOMAC

Postoperatively, WOMAC scores decreased over time until the last follow‐up, when WOMAC scores significantly reduced from 62.38 ± 17.90 preoperatively to 20.38 ± 13.75, with significant improvements in WOMAC scores at all follow‐up time points compared with preoperative ones (Table S3 and Figure 4C). At the final follow‐up, seven patients had elevated WOMAC scores, five of whom were over 50 years of age. Correlation analysis showed no significant correlation between ΔWOMAC and onset time, age, gender, follow‐up time, side of onset, or imaging grading.

### 3.5 Lysholm Knee Score

Postoperatively, the Lysholm Knee Score increased over time until the last follow‐up, when it increased significantly from 36.38 ± 11.21 points preoperatively to 81.39 ± 12.32 points at all follow‐up time points compared with preoperatively (Table S3 and Figure 4D). At the final follow‐up, Lysholm scores decreased in three patients, all over 50 years of age. Correlation analysis showed no significant correlation between ΔLysholm Knee Score and onset time, age, gender, follow‐up time, side of onset, or imaging grading.

### 3.6 Mobility

Postoperatively, changes in knee mobility were observed over time until the last follow‐up, when the knee extension angle changed from 11.92° ± 14.94° to 0.00° ± 0.00° preoperatively and the knee flexion angle changed from 122.69° ± 10.9° to 133.08° ± 4.45° preoperatively(*p* < 0.05). No statistically significant difference was found in the knee extension angle compared to preoperative at all follow‐up time points, whereas there was a significant improvement in the knee flexion angle. Correlation analysis showed no significant correlation between mobility and time of onset, age, gender, follow‐up time, side of onset, or imaging grading.

### 3.7 Subjective Satisfaction of Patients With Surgical Outcomes

During the last follow‐up, we investigated the satisfaction level of all patients with the surgical outcomes. More than half of the patients expressed satisfaction with the surgical effect (53.85%), and the remaining patients all indicated that they were relatively satisfied with the surgical results.

## 4 Discussion

This study retrospectively included 13 patients suffering from knee and synovial chondromatosis to determine the efficacy of arthroscopic knee cleanup surgery. All the patients showed significant pain relief after knee surgery and considerable knee mobility and function improvement. Adverse events occurred after the enrollment of patients in this study. No local or systemic complications were observed, and no malignant transformation has been observed. Our results showed that age, follow‐up time, side of onset, and preoperative imaging evaluation grading did not affect the degree of postoperative pain relief and functional improvement of the knee. The survey results of patients' subjective satisfaction with the clinical outcomes of the surgery during the last follow‐up also indicated that patients were generally satisfied with the surgical results, meeting their expectations. The preoperative VAS score and its change (ΔVAS score) exhibit a positive correlation with the onset time, suggesting that patients who experience a longer duration of symptoms prior to surgery tend to have more pronounced baseline pain levels. Consequently, these patients may also exhibit more substantial improvements in postoperative pain relief. Nevertheless, caution is advised in drawing this conclusion, as the KOOS pain subscale did not demonstrate a similar trend. Our statistical analysis indicates a potential association between gender and two subscales of the KOOS questionnaire: the symptom subscale and the exercise subscale. In our study, males demonstrated higher average change (Δ) values in these two scales compared to females. Furthermore, the analysis of the KOOS motor function and quality of life subscales suggests a positive correlation between the onset time of symptoms and the extent of postoperative recovery in motor function, as well as the enhancement of quality of life. This implies that patients with a longer duration of symptoms prior to surgery may achieve a better recovery in exercise capacity and an overall improvement in their quality of life. However, it is important to note that the changes observed in WOMAC and Lysholm scores did not indicate any significant relationship with either the onset time or gender, so these conclusions should be treated with caution.

Furthermore, our analysis focused on the variations in scores at consecutive follow‐up intervals. The findings revealed that, with the exception of the initial baseline assessment and the 3‐month postoperative mark, the changes in pain and function scores between other adjacent time points were not statistically significant. This observation could be attributed to the fact that by this juncture, the immediate trauma and inflammation typically associated with surgery would have largely resolved, thereby allowing the therapeutic benefits of the surgery to become fully manifest and contribute to the amelioration of patients' symptoms.

### 4.1 Potential Factors Influencing Surgical Efficacy

Synovial chondromatosis has been found in 33 different locations throughout the body, including the knee, hip, ankle, and elbow, with the knee having the highest incidence [25]. Synovial chondromatosis is challenging to diagnose, with an average time interval from symptom onset to reaching a diagnosis of 30–38 months [26, 27], and in our study, the time of onset ranged from 2 to 96 months, with an average of 43.55 months required to reach a precise diagnosis. Although some studies have reported that delayed diagnosis may lead to the progression of osteoarthritis [28], our findings did not note that the time to onset was significantly associated with patients' preoperative pain and functional scores, nor did it significantly affect patients' postoperative pain and operational improvement. In addition to the typical history and signs, imaging is integral to diagnosing accurately. Free bodies caused by synovial chondromatosis can be ossified or non‐ossified, and X‐rays can show sparse or calcified bodies. Still, if the nodes lack calcification, synovial chondromatosis may not be detected in up to 30% of cases [29]. Therefore, MRI examination becomes very important in patients with synovial chondromatosis and also allows better assessment of the extent of joint damage; studies have reported that synovial chondromatosis usually shows low to moderate signal intensity on t1‐weighted images and high signal intensity on t2‐weighted images, with low‐signal calcification on both images. The characteristics of the nodules may vary depending on the stage of formation [30]. In this study, the degree of cartilage damage was also graded by imaging, and the results showed a significant positive correlation between age and Kellgren and Lawrence grading and Recht grading; however, postoperative follow‐up showed that patients' pain relief and functional improvement were independent of imaging grading and that patients with varying degrees of cartilage damage benefited from arthroscopic‐free body resection. Patients with varying degrees of cartilage damage can benefit from arthroscopic‐free body resection. In conclusion, our study shows that patients of different sexes, laterality, time of onset, and imaging grading will benefit from synovial chondromatosis surgery in the long term. This could be due to knee arthroscopy directly addressing the core pathological issues of pain and function recovery, thus reducing the impact of baseline variables. However, our findings are limited by the study design. Future validation requires larger, stratified studies.

### 4.2 Observation on the Long‐Term Therapeutic Effect of Arthroscopic Surgery for Synovial Chondromatosis of the Knee

Synovial chondromatosis may lead to symptoms such as pain and limited joint mobility and have the potential for malignant transformation. Past studies have worked to address this issue, with a study by Murphy et al. reporting 32 patients treated with open surgery to remove the free body and synovium, with only 1 patient experiencing a recurrence after surgery [31]. As arthroscopic techniques have become more widely available, arthroscopic debridement combined with synovectomy appears to be more effective than open surgery. This may be attributed to the fact that arthroscopic synovectomy can accurately identify and remove the affected synovium. However, the arthroscopic technique demands a high level of skill from the surgeon, and there is a potential risk of injury due to the proximity of blood vessels to the posterior capsule [32]. The results of a survey by Ogilvie‐Harris et al. showed a significant difference in the recurrence rate between arthroscopic resection of the free body and arthroscopic synovectomy, with all recurrences subsequently being successfully treated with arthroscopic synovectomy [33]. Synovectomy is generally recommended, although there is still controversy regarding the effectiveness of performing synovectomy to prevent recurrence [34, 35]. All of our patients underwent arthroscopic‐free body resection and synovectomy, and no recurrence of clinical symptoms was observed. This finding is consistent with the previously published findings of Ogilvie‐Harris et al. During more than 5 years of follow‐up, our patients experienced significant improvement in knee pain and function after arthroscopic surgery, and maintained their pain improvement and functional recovery better during long‐term follow‐up. Although some patients showed changes in functional scores, most knees were over 50 years of age, possibly due to the effects of knee osteoarthritis. Secondary synovial chondromatosis may result from osteoarthritis, and prolonged synovial chondromatosis can lead to irreversible damage to the cartilage and joints, ultimately contributing to the progression of osteoarthritis [14]. All patients included in this study were diagnosed with knee osteoarthritis at the time of their synovial chondromatosis diagnosis. Although arthroscopic removal of synovial chondromatosis can delay the progression of osteoarthritis [36], one patient in this study with a preoperative K‐L grade of 3 progressed to end‐stage osteoarthritis of the knee at the time of the final follow‐up and required knee replacement, due to the lack of systematic postoperative osteoarthritis of the knee treatment and rehabilitation exercises.

### 4.3 Limitations and Strengths

There are limitations to our results. First, because we conducted a retrospective study, it introduces unavoidable recall bias and the possible effects of unnoticed bias and confounding factors. Second, due to the lower prevalence of synovial chondromatosis of the knee, we had a relatively small number of included cases, which may have affected the reliability of the results and led to difficulties in further stratified analyses. Future multicenter retrospective or prospective registry data will help validate these promising findings. In addition, the lack of a control group limited our ability to determine whether the research results were due to the intervention measures themselves or the natural progression of the disease. Our study used preoperative and postoperative results of patients to compare and compensate for this deficiency. It thus needed to eliminate the influence of extraneous variables on the study results. Ultimately, the applicability of our research findings is confined to patients who have undergone surgical treatment for the first time. For individuals with a history of prior knee joint surgery, coexisting knee joint pathologies, or severe systemic conditions, additional studies are warranted to validate our findings in these specific patient populations.

However, our findings demonstrate some of our experience in the treatment of synovial chondromatosis of the knee; we are convinced that arthroscopic surgery represents a superior treatment option for patients with synovial chondromatosis. Compared to open surgery, it causes less patient trauma, allows faster recovery, and delivers obvious clinical benefits.

The primary strength of our research lies in its pioneering observation of the long‐term efficacy of arthroscopic treatment for synovial chondromatosis. We have meticulously compared multiple critical indicators, including patient activity levels, functional scores, and overall satisfaction, to comprehensively assess the extent of symptom relief and functional improvement following surgery. Notably, our findings suggest that factors such as gender, onset time, and imaging grading do not significantly influence the therapeutic outcomes of this procedure. These insights are valuable in guiding the selection of clinical treatment options and could serve as a foundation for the design of future prospective studies with larger sample sizes. Future studies might stratify outcomes by severity of osteoarthritis at baseline (e.g., K‐L grade) to better identify predictors of long‐term arthroscopic success. The potential impact of postoperative rehabilitation on long‐term outcomes may also be a focus of future research.

## 5 Conclusion

This retrospective study demonstrated that arthroscopic‐free body resection and synovectomy is an effective treatment modality for patients with synovial chondromatosis of the knee. All of the patients obtained significant pain relief and functional improvement after the procedure, and the effectiveness of the treatment was not significantly affected by the age, time of onset, gender, side of onset, and imaging grading of the patients. Compared with previous studies, our study focused more on the long‐term clinical efficacy of arthroscopic resection of synovial chondromatosis, and the results of more than 5 years of follow‐up showed that all patients had no recurrence of clinical symptoms, and the pain relief and functional recovery were maintained well.

## Author Contributions

All authors contributed to the study conception and design. All authors are in agreement with the manuscript. Y.X. and T.L. were responsible for the conceptualization of the project, data management, formal analysis, design of methodology, and the survey process. L.Y. and W.M. participated in the survey follow‐up and visualization of the study. J.L. provided resources, obtained funding, and conducted project management and supervision. Y.X. drafted the initial manuscript of this study, and T.L. and J.L. were in charge of reviewing and editing the article.

## Ethics Statement

This study has been approved by the Biomedical Ethics Review Committee of West China Hospital of Sichuan University with approval number 2023 (2398).

## Consent

Informed consent was obtained from all individual participants included in the study. The authors affirm that human research participants provided informed consent for publication of the images in Figure 2.

## Conflicts of Interest

The authors declare no conflicts of interest.

## Supporting information


**TABLE S1.** Patient baseline characteristics and clinical outcomes.
**TABLE S2:** Comparison of patients’ VAS before and after surgery ($\bar{x}+s$).
**TABLE S3:** Comparison of patients’ functional score before and after surgery ($\bar{x}+s$).

## Data Availability

The datasets used and/or analyzed during the current study are available from the corresponding author on reasonable request.
