# Supplementary material for: Efficacy Analysis of Arthroscopic Treatment of Synovial Chondromatosis of the Knee: A Retrospective Study of More Than Five Years
Source: Orthop Surg. 2025 Jul 21;17(9):2608–16. doi: 10.1111/os.70132 (PMC12404863; doi:10.1111/os.70132)
Supplement: Supplementary file 1 — TABLE S1. Patient baseline characteristics and clinical outcomes. TABLE S2: Comparison of patients’ VAS before and after surgery (x¯+s). TABLE S3: Comparison of patients’ functional score before and after surgery (x¯+s). [file OS-17-2608-s001.docx]

Supplementary files 1 ：Patient baseline characteristics and clinical outcomes

| Parameter | Value/Distribution |
| --- | --- |
| Total cases | 13 patients |
| Gender | Male: 2 4; Female: 9 |
| Age (years) | 44.32 ± 16.10 (range 18.2-63.8) |
| Follow-up duration (months) | 113.15 ± 30.45 months (range 61-145) |
| Disease duration (months) | 47.00 ± 34.10 months (range 2-96) |
| Affected side | Left: 8 ; Right: 5 ; Bilateral: 1 |
| Postoperative complications | Infections, neurovascular complications, malignant transformation, or symptom recurrence: 0 cases |
| Kellgren-Lawrence grading | Grade 1: 7 ; Grade 2: 2 ; Grade 3: 4 ; Grade 4: 0 |
| Recht cartilage injury grading | Grade 1: 3 ; Grade 2: 4 ; Grade 3: 3 ; Grade 4: 3 ; Grade 5: 0 |

Supplementary files 2: Comparison of patients' VAS before and after surgery（±s）

| VAS | Mean±SD | P-value (compared to preoperative) |
| --- | --- | --- |
| Preoperative | 7.23±1.54 |  |
| 3 months postoperative | 2.46±1.05 | <0.0001 |
| 6 months postoperative | 1.46±0.52 | <0.0001 |
| 1 year postoperative | 1.15±0.80 | <0.0001 |
| 5 years postoperative | 1.31±0.95 | <0.0001 |
| Last follow-up | 1.77±1.48 | <0.0001 |

Supplementary files 3: Comparison of patients' functional score before and after surgery（±s）

| Functional score | Mean±SD | P-value (compared to preoperative) |  |
| --- | --- | --- | --- |
| KOOS pain subscale | | |  |
| Preoperative | 39.85±9.31 |  |  |
| 3 months postoperative | 14.77±5.17 | <0.0001 |  |
| 6 months postoperative | 8.92±4.60 | <0.0001 |  |
| 1 year postoperative | 8.54±4.88 | <0.0001 |  |
| 5 years postoperative | 8.08±4.29 | <0.0001 |  |
| Last follow-up | 9.52±6.59 | <0.0001 |  |
| KOOS symptoms subscale | | |  |
| Preoperative | 47.15±19.55 |  |  |
| 3 months postoperative | 13.77±8.18 | <0.0001 |  |
| 6 months postoperative | 11.08±7.33 | <0.0001 |  |
| 1 year postoperative | 10.54±7.36 | <0.0001 |  |
| 5 years postoperative | 9.77±6.73 | <0.0001 |  |
| Last follow-up | 12.92±7.87 | <0.0001 |  |
| KOOS activities of daily living subscale | | |  |
| Preoperative | 33.69±7.58 |  |  |
| 3 months postoperative | 16.54±5.21 | <0.0001 |  |
| 6 months postoperative | 10.92±5.16 | <0.0001 |  |
| 1 year postoperative | 9.08±4.96 | <0.0001 |  |
| 5 years postoperative | 9.00±5.54 | <0.0001 |  |
| Last follow-up | 10.69±7.89 | <0.0001 |  |
| KOOS exercise subscale | | | |
| Preoperative | | 56.92±14.07 |  |
| 3 months postoperative | | 31.92±13.93 | <0.0001 |
| 6 months postoperative | | 24.54±13.27 | <0.0001 |
| 1 year postoperative | | 22.69±13.94 | <0.0001 |
| 5 years postoperative | | 21.92±13.77 | <0.0001 |
| Last follow-up | | 22.78±15.13 | <0.0001 |
| KOOS quality of life subscale | | | |
| Preoperative | | 57.38±17.02 |  |
| 3 months postoperative | | 25.94±10.95 | <0.0001 |
| 6 months postoperative | | 19.46±7.87 | <0.0001 |
| 1 year postoperative | | 19.46±7.87 | <0.0001 |
| 5 years postoperative | | 20.38±8.49 | <0.0001 |
| Last follow-up | | 23.23±13.71 | <0.0001 |
| WOMAC | | | |
| Preoperative | | 62.38±17.90 |  |
| 3 months postoperative | | 26.08±9.80 | <0.0001 |
| 6 months postoperative | | 19.38±9.80 | <0.0001 |
| 1 year postoperative | | 16.92±8.83 | <0.0001 |
| 5 years postoperative | | 16.85±10.59 | <0.0001 |
| Last follow-up | | 20.38±13.75 | <0.0001 |
| Lysholm Knee Score | | | |
| Preoperative | | 36.38±11.21 |  |
| 3 months postoperative | | 76.85±8.33 | <0.0001 |
| 6 months postoperative | | 83.00±7.99 | <0.0001 |
| 1 year postoperative | | 84.92±7.92 | <0.0001 |
| 5 years postoperative | | 84.23±9.16 | <0.0001 |
| Last follow-up | | 81.39±12.32 | <0.0001 |
